# Inhibition studies of the protozoan α-carbonic anhydrase from *Trypanosoma cruzi* with phenols

**DOI:** 10.1080/14756366.2022.2119965

**Published:** 2022-09-06

**Authors:** Alessandro Bonardi, Seppo Parkkila, Claudiu T. Supuran

**Affiliations:** a Neurofarba Department, Pharmaceutical and Nutraceutical Section, University of Florence, Sesto Fiorentino (FI), Italy; b Faculty of Medicine and Health Technology, Tampere University, Tampere, Finland; c Fimlab Ltd, Tampere University Hospital, Tampere, Finland

**Keywords:** Carbonic anhydrase, anti-protozoal action, phenol, *Trypanosoma cruzi*, enzyme inhibition

## Abstract

The α-class carbonic anhydrase (CA, EC 4.2.1.1) from the protozoan pathogen *Trypanosoma cruzi*, TcCA, was investigated earlier for its inhibition with anions, sulphonamides, thiols and hydroxamates, well-known classes of CA inhibitors (CAIs). Here we present the first inhibition study of this enzyme with phenols, which possess a diverse CA inhibition mechanism compared to the previously investigated compounds, which are all zinc binders. Indeed, phenols are known to anchor to the zinc coordinated water molecule within the enzyme active site. In a series of 22 diversely substituted phenols, the best inhibitors were simple phenol, pyrocatechol, salicylic acid, 3,5-difluorophenol, 3,4-dihydroxy-benzoic acid, 3,6- dihydroxy-benzoic acid, caffeic acid and its des-hydroxy analog, with K_I_s of 1.8 − 7.3 µM. The least effective TcCA inhibitor was 3-chloro-4-amino-phenol (*K_I_* of 47.9 µM). Although it is not yet clear whether TcCA can be considered as an anti-Chagas disease drug target, as no animal model for investigating the antiprotozoan effects is available so far, finding effective *in vitro* inhibitors may be a first relevant step towards new antiprotozoal agents.

## 1. Introduction

Protozoans are microscopic, nonfilamentous protists belonging to a multitude of phyla, with many genera and species described so far, many of which possess ecological and industrial relevance. However, they sometimes produce disease in vertebrates, which may range from mild to moderate, such as those induced by *Toxoplasma gondii* or *Entamoeba histolytica*, or may lead to more serious conditions, in the case of infections due to *Cryptosporidium parvum*, *Giardia lamblia*, *Trichomonas vaginalis*, *Babesia* spp., but also very serious and widespread ones, such as malaria, leishmaniasis, Chagas disease, and African sleeping disease^1^^,^^2^. Although rare, there are also several fatal protozoal diseases, mostly provoked by amoebae belonging to *Naegleria fowleri, Acanthamoeba* spp. and *Balamuthia mandrillaris* genera/species^1^. Few effective therapeutic approaches are available so far for treating most diseases provoked by protozoans^1^. Albeit all 12 protozoans genera which produce human disease are well studied by now, there are few drugs useful for treating them. Furthermore, these drugs have been available for many decades, generally show high toxicity and low therapeutic indexes, and more concerning, extensive resistance to these treatment options has developed in the last period^1^^,^^2^.

Among the prozoan diseases which drew much attention in the last decade is Chagas’s disease (CD), provoked by *Trypanosoma cruzi*, a pathogen thought to be endemic to South America, but which is nowadays also infecting people in Europe and North America^3–7^. This parasite possess an intricate life cycle, with many growth stages, not all of which are sensitive to the two clinically used drugs, nifurtimox and benznidazole, both of them belonging to the nitro-azole, old class of antiprotozoal drugs^1^^,^^2^^,^^7^. Thus, there is a stringent need of new drug targets for fighting CD, and although many of them have been proposed so far^2^, no relevant progress has been achieved for the moment^7^.

An α-class carbonic anhydrase (CA, EC 4.2.1.1) has been identified, cloned and characterised in the genome of *T. cruzi* few years ago by our groups^3^. This enzyme, denominated TcCA, was shown to possess high catalytic activity for the conversion of CO_2_ into bicarbonate and protons^3^, was also shown to be inhibited, sometimes quite efficiently, by the main classes of CA inhibitors (CAIs), such as the anions, sulphonamides, thiols and hydroxamates^3–6^. In some cases interesting antiprotozoal effects were also observed *ex vivo* in cell cultures with some of them, e.g. hydroxamates and sulphonamides formulated as nanoemulsions^6^^,^^7^. It is not yet definitely clear whether TcCA can indeed be considered as an anti-CD drug target, since no animal model for investigating the antiprotozoan effects is available so far^1^^,^^2^. However, the interesting *in vitro* and *ex vivo* data available with many classes of CAIs (not all of which possess the optimal pharmacological properties, such as for example a facile membrane penetration^6^) prompts us to continue the investigation of new classes of inhibitors targeting this pathogenic enzyme. Here we report the first inhibition study of TcCA with a series of phenols, well-known inhibitors of CAs^8–11^.

## 2. Materials and methods

### 2.1. Enzymology and CA activity and inhibition measurements

Production and purification of recombinant TcCA have been previously described by our groups^3^. An Applied Photophysics stopped-flow instrument was used to assay the CA- catalysed CO_2_ hydration activity^12^. Phenol red (0.2 mM) was used as a pH indicator, working at the absorbance maximum of 557 nm, with 10 mM HEPES (pH 7.4) as a buffer, and in the presence of 10 mM NaClO_4_ to maintain constant ionic strength, in order to follow the initial rates of the CA-catalysed CO_2_ hydration reaction for a period of 10–100 s. The CO_2_ concentrations ranged from 1.7 to 17 mM for the determination of the kinetic parameters and inhibition constants. TcCA concentration in the assay system was 10.6 nM. For each inhibitor, at least six traces of the initial 5–10% of the reaction were used to determine the initial velocity. The uncatalyzed rates were determined in the same manner and subtracted from the total observed rates. Stock solutions of inhibitors (10–20 mM) were prepared in distilled-deionized water, and dilutions up to 10 nM were done thereafter with the assay buffer. Inhibitor and enzyme solutions were preincubated together for 15 min prior to the assay, in order to allow for the formation of the E-I complex. The inhibition constants were obtained by non-linear least-squares methods using Prism 3 and the Cheng-Prusoff equation, as reported previously^3^^,^^4^, and represent the mean from at least three different determinations.

### 2.2. Chemistry

Compounds **1–22**, buffers, acetazolamide **AAZ** and other reagents were of > 99% purity and were commercially available from Sigma-Aldrich (Milan, Italy).

## 3. Results and discussion

CAs possess several classes of inhibitors which interact with the enzyme in a rather intricate and sometimes unexpected way^13–16^. Indeed, the classical inhibitors, such as the inorganic/organic anions, as well as the sulphonamides and their isosteres (sulfamides and sulfamates) coordinate to the catalytic metal ion, which is crucial for catalysis, and substitute the coordinated nucleophilic water molecule/hydroxide ion^13^^,^^14^. However, many classes of CAIs identified more recently, such as the phenols, polyamines, sulfocoumarins, alcohols, etc., interact with the enzyme in diverse modes, inhibiting it by anchoring to the metal-ion coordinated water molecule^15^^,^^16^, obstructing the entrance to the active site cavity^17^^,^^18^, or even binding outside the active site^19^. In particular, phenols, polyphenols, alcohols and more recently β-mercapto-ethanol^13^^,^^16^ were shown by X-ray crystallography to anchor with their OH moiety by means of hydrogen bond(s) to the zinc coordinated water molecule (eventually making other strong interactions with amino acid residues in the neighbourhood of the catalytic core) in α-, β- and γ-CAs, making this inhibition mechanism a quite general one^13^^,^^14^. Initially, Lindskog’s group^8^ reported phenol to act as a weak CAI, whereas Christianson’s group then resolved the X-ray crystal structure of this compound bound to the human(h) isoform hCA II^9^. Since then, many synthetic and natural phenols/polyphenols were investigated for their interaction with many CAs of diverse origin, leading to the discovery of interesting leads^10^^,^^11^^,^^20–24^.

Considering the wealth of literature data on inhibition of various CAs from mammals and pathogenic organisms with phenols, and the lack of such studies for the inhibition of TcCA, here we report the inhibition of this enzyme with a library of 22 phenols (Table 1) investigated earlier for their interaction with human, bacterial and plasmodial CAs^10–24^. The following structure activity relationship (SAR) can be evidenced from the inhibition data presented in Table 1, in which the hCA I and II inhibition data are also provided for comparison reasons:

**Table 1.** Inhibition data of human CA isoforms I and II and protozoan enzyme TcCA by a stopped-flow CO_2_ hydrase assay method [12] using the sulphonamide acetazolamide (**AAZ)** as standard drug

Name	Structure	*K_i_* (µM)^a^		
		hCA I	hCA II	TcCA
**1**	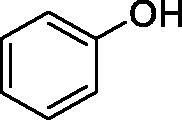	10.2	5.5	3.4
**2**	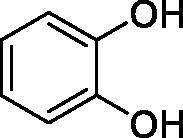	>100	5.5	2.1
**3**	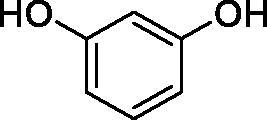	>100	9.4	32.7
**4**	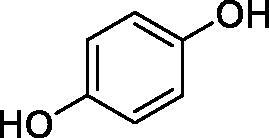	10.7	0.1	18.5
**5**	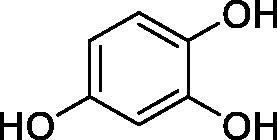	>100	>100	13.2
**6**	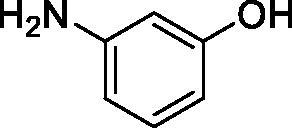	4.9	4.7	41.7
**7**	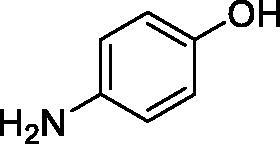	>100	>100	25.3
**8**	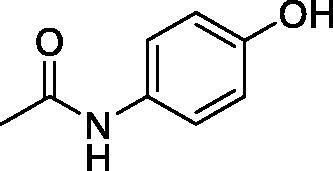	10.0	6.2	19.8
**9**	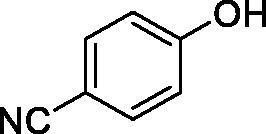	>100	0.1	15.1
**10**	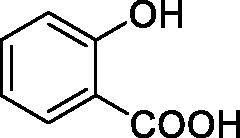	9.9	7.1	4.5
**11**	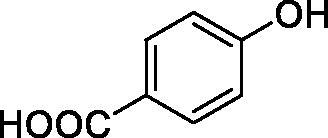	9.8	10.6	28.4
**12**	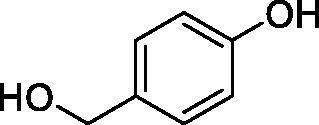	68.9	95.3	17.8
**13**	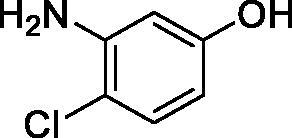	6.3	4.9	13.6
**14**	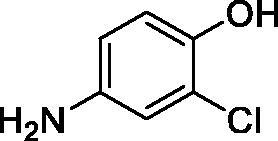	57.8	57.5	47.9
**15**	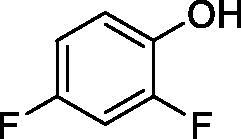	>100	>100	21.1
**16**	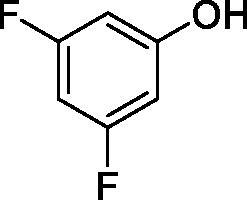	38.8	33.9	7.3
**17**	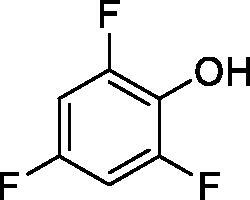	>100	>100	26.9
**18**	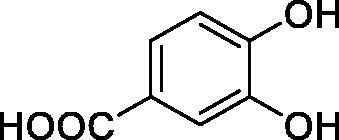	1.1	0.5	2.4
**19**	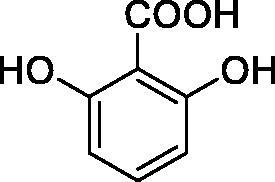	5.7	5.2	15.9
**20**	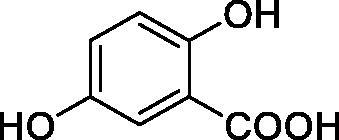	4.2	4.1	7.1
**21**	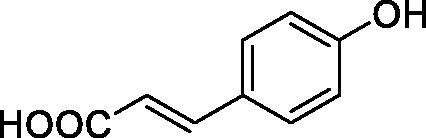	1.1	1.3	4.8
**22**	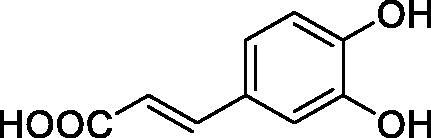	2.4	1.6	1.8
**AAZ**	–	0.25	0.012	0.06

^a^Mean from three different assays, by a Stopped-Flow technique (errors were in the range of ± 5–10% of the reported values).

- All phenols investigated here of types **1–22** inhibited TcCA with K_I_s in the micromolar range, more precisely of 1.8–47.9 µM. It should be noted that the investigated compounds incorporate one, two or three phenolic OH moieties, and generally one two or three other simple substituents, of the amino, hydroxyl, halogeno, carboxy, cyano, acetamido or hydroxymethyl type. Few of them (**21** and **22**) possess the carboxyethenyl moiety which is slightly bulkier compared to the moieties present in the other scaffolds of type 1-20 (Table 1).
- The most effective TcCA inhibitors were **1, 2, 10, 16, 18** and **20-22**, with K_I_s in the range of 1.8–7.3 µM. They include the simple phenol **1**, pyrocatechol **2**, salicylic acid **10**, 3,5-difluorophenol **16**, 3,4-dihydroxy-benzoic acid **18**, 3,6- ihydroxy-benzoic acid **20** as well as caffeic acid **22** and its des-hydroxy analog **21**. The best inhibitor was just caffeic acid **22** (*K_I_* of 1.8 µM) as well as pyrocatechol **2** (*K_I_* of 2.1 µM). It should be noted that caffeic acid in fact incorporates in its molecule the pyrocatechol fragment, also present in **18** (the next most effective inhibitor in this series). However, this fragment not always induced the most effective inhibitory power, as in compound **5**, it only led to a moderate inhibitor (*K_I_* of 13.2 µM).
- Slightly weaker TcCA inhibitory effects compared to the compounds discussed above were observed for the following phenols: **4, 5, 8, 9, 12, 13**, and **19**, which possessed K_I_s in the range of 13.2–19.8 µM. The structure activity relationship (SAR) is again not easy, since apart 19, which is a 2,6-dihydroxy-substituted phenol, the other derivatives generally have a 4-substituent, of the OH, CN, acetamido, hydroxymethyl or Cl type. Thus, the structural diversity is rather high in order to draw straightforward SAR conclusions.
- The least effective TcCA inhibitors were **3, 6, 7, 11, 14, 15**, and **17**, which showed K_I_s in the range of 21.1–47.9 µM. As mentioned above, also these derivatives possess a heterogeneous structure which makes SAR discussions not easy to interpret. Howeve, it seems that the presence of amino groups in *meta* or *para* to the phenol functionality (as in **6** and **14**) was associated with weaker TcCA inhibitory properties. In fact these two compounds are the least effective inhibitors (K_I_s of 41.7–47.9 µM). The presence of a chlorine in *para* (in addition to the amino in *meta*) however increased the inhibitory power, since compound **13** was a more effective TcCA inhibitor (*K_I_* of 13.6 µM) compared to **14**. These two compounds are position isomers, which demonstrates that even small structural changes may lead to dramatic differences in the inhibitory power.
- TcCA has a very diverse inhibition profile with phenols compared to the human isoforms hCA I and II, for which these compounds showed very diverse K_I_s (Table 1). However, all phenols are much weaker CAIs compared to the sulphonamide acetazolamide, which is a nanomolar inhibitor for all three enzymes.

## 4. Conclusions

Recently, several groups showed that the inhibitors of bacterial CAs may lead to effective compounds for fighting drug resistant bacteria^24–26^, although there was some relevant scepticism that these enzymes could be considered as antiinfective drug targets^27^. It took more than 10 years since the first proposal that bacterial CAs may be new drug targets for the development of antibiotics^28^ till the actual *in vivo* validation of some of them, many of which present in relevant and drug resistant bacterial pathogens, such as *Enterococci*, *Neisseria* spp., *Helicobacter pylori*, etc^25^^,^^26^. This was only possible through a dedicated medicinal chemistry approach for developing new CAIs selective for the bacterial over the human enzymes, but also due to the development of animal models of such bacterial diseases in which many of these compounds were tested^26^. In the case of the protozoan CAIs, although there are plenty of effective and rather selective *in vitro* inhibitors, there is a lack of animal models of most such infections, partly due to the complicated life cycles of these pathogens. This is particularly true in the case of CD: *T. cruzi* has two evolutive forms, with the first one being the circulating infective but not replicative form, known as trypomastigotes, and the second one being the replicative, intracellular form, known as amastigotes, which have also been shown to be infective^1^^,^^2^. Thus, as long as there will be impossible to test the efficacy of newly designed enzyme inhibitors, as those investigated here, on both evolutive forms of *T. cruzi* it is difficult to estimate the real contribution of protozoan CAs in the pathogenicity and infectiveness of these protozoa.
